# Impact of Systemic Medications on Oral Health: A Cross-Sectional Study Among Geriatric Patients

**DOI:** 10.7759/cureus.88964

**Published:** 2025-07-29

**Authors:** Avina Kharat, Omkar Prasad Baidya, Sourav Hambirrao Choudhari, Nasim Vahid Shakeela, Rasha Nasim, Kalpesh Hathi

**Affiliations:** 1 Department of Pharmacology, Employees' State Insurance Corporation (ESIC) Medical College and Hospital, Indore, IND; 2 Department of Physiology, Jagannath Gupta Institute of Medical Sciences and Hospital, Kolkata, IND; 3 Department of Surgery, Government Medical College, Kozhikode, Kozhikode, IND; 4 Department of Pedodontics, Government Dental College, Thiruvananthapuram, Thiruvananthapuram, IND; 5 Department of Orthodontics and Dentofacial Orthopedics, PMS College of Dental Science and Research, Thiruvananthapuram, IND; 6 Department of Pathology, Matushri Prabhaben Khodabhai Boghara Medical College and Research Centre and Hospital, Atkot, IND

**Keywords:** aging population, geriatric oral health, polypharmacy, systemic medications, xerostomia

## Abstract

The increasing prevalence of chronic diseases in the aging global population has led to widespread use of systemic medications, often resulting in polypharmacy among geriatric patients. While these drugs are essential for managing conditions such as hypertension, diabetes, cardiovascular diseases, and autoimmune disorders, their adverse effects on oral health remain underrecognized and insufficiently studied. This review synthesizes current literature alongside cross-sectional observational data to evaluate the multifaceted impact of commonly prescribed systemic medications on oral health outcomes in elderly populations. Key oral manifestations include xerostomia, gingival overgrowth, mucosal ulcerations, medication-related osteonecrosis of the jaw, bleeding complications, taste alterations, and increased susceptibility to infections. The interplay between age-related physiological changes, polypharmacy, and systemic disease complicates oral care and exacerbates morbidity. Despite the clinical significance, oral side effects are rarely emphasized in pharmacological guidelines, and geriatric-specific data remain limited. The review highlights the need for interdisciplinary collaboration between medical and dental professionals, comprehensive geriatric oral health screening protocols, and increased clinician awareness to improve management and preventive strategies. This integrated approach is essential to enhance the quality of life and oral health outcomes in elderly patients receiving long-term systemic pharmacotherapy.

## Introduction and background

As the world’s population ages, healthcare systems everywhere are being strongly affected. According to the World Health Organization, the number of people aged 60 and older is expected to be more than double by 2050, reaching over two billion [1]. Due to this shift, the number of chronic non-communicable diseases, for example, hypertension, diabetes, heart conditions, and neurodegenerative disorders, has increased, and all of them need long-term treatment with medications [2].

For this reason, older adults are commonly given several medications at the same time, which is called polypharmacy [3]. In different clinical settings and regions, between 30% and 70% of elderly patients may be affected by polypharmacy [4]. While these therapies are often needed to treat diseases, they can cause many side effects, including problems with oral health.

Even though maintaining oral health is essential for overall well-being and quality of life, it is often overlooked in the care of elderly individuals. As people age, their saliva production decreases, their immune system becomes weaker, tissues heal more slowly, and the structure of the mouth changes [5]. As people age, they are more likely to experience dental caries, gum disease, lose their teeth, mouth infections, and oral discomfort [6]. In addition, many seniors have difficulties with their hands, thinking, and money, which all make it more challenging to maintain good oral hygiene. When systemic medication side effects are added to these natural vulnerabilities, it results in much more oral disease, but this connection is often missed in regular clinical care [7].

Various and intricate ways are used by systemic medications to affect oral health. Many medicines, for example, anticholinergics, antidepressants, and antihypertensives, can cause xerostomia [8]. Having xerostomia not only makes your mouth uncomfortable but also weakens the protection saliva offers against dental decay, fungal infections, and periodontal problems [9]. Taking calcium channel blockers or anticonvulsants can result in overgrowth of the gums, which can be difficult to clean and raises the risk of periodontitis [2]. Using immunosuppressants such as cyclosporine, tacrolimus, and azathioprine, and chemotherapeutic agents like methotrexate, 5-fluorouracil, and doxorubicin may cause ulcers in the mouth, slow healing, and increase the risk of infection. Additionally, bisphosphonates (e.g., zoledronic acid, alendronate) and certain monoclonal antibodies (e.g., denosumab) have been associated with a serious condition called medication-related osteonecrosis of the jaw (MRONJ) [5]. Since these drugs are commonly given to this group, dental procedures can be challenging because they may cause prolonged bleeding [10].

Even though these effects are important, oral issues are often overlooked in drug safety information and guidelines for doctors. Because many healthcare workers do not receive training on oral complications, patients may not receive the best care [11]. Many geriatric patients are not included in clinical trials, so there is not much data on the oral effects of systemic medications for this group [12]. Since there are gaps in information and practice, a better integrated and interdisciplinary approach is needed to manage the health of older adults on long-term medications.

While it is well known that systemic drugs can affect the mouth, most studies look at individual drug groups or particular oral problems. It is difficult to find reviews that bring together all these findings into a single framework suitable for older adults. Most of the evidence comes from studies of general adult groups, so it is less useful for the elderly, who have different physiological, pharmacokinetic, and functional characteristics. There is an urgent need for a review that links geriatric medicine, pharmacology, and dental science.

Objectives of the review

This article is designed to review the effects of common systemic medications on the mouths of elderly people. It deals with a wide range of drugs such as those used for heart, hormone, brain, immune system, and cancer problems. Special focus is placed on the causes of drug-related oral problems, how they are recognized, and how they are managed. Besides summarizing present research, the article adds insights from an observational analysis to make the review more applicable to real-life clinical situations. This review suggests that by paying attention to the many ways medications and oral health are related in the elderly, healthcare providers should work together and include oral health in the care of chronic diseases.

## Review

Methodological considerations

In order to gather a wide variety of research discussing the effects of systemic drugs on oral health in geriatric populations, a systematic search of pertinent literature was carried out using the databases PubMed, Scopus, and Web of Science. In order to narrow down the results, the search method used Boolean operators in conjunction with keywords and Medical Subject Headings (MeSH), including "geriatric oral health," "systemic drugs," "polypharmacy," "xerostomia," "gingival hyperplasia," and "medication-related osteonecrosis." In order to guarantee relevance to the senior population, inclusion criteria were developed to concentrate on human research published in English between 2000 and 2025, specifically involving participants 60 years of age and older. The review prioritized papers that examined drug-induced oral problems, the effects of polypharmacy, and pharmacological influences on salivary function. It took into account a variety of study methods, including systematic reviews, cross-sectional studies, clinical trials, and case series. After abstract and full-text screening, studies that were solely focused on geriatric populations, published in languages other than English, or lacked methodological rigor were eliminated. Emphasis was placed on high-quality evidence to inform the synthesis of current knowledge, with data extracted on medication types, oral health outcomes, mechanisms of action, and clinical significance. This method allowed for a comprehensive and current summary of the intricate relationships between systemic drugs and oral health in senior citizens, which served as the foundation for the thematic review that is discussed in the parts that follow.

Polypharmacy and oral health in the elderly

Many elderly people use five or more medications at once due to multiple chronic conditions, including cardiovascular disease, diabetes, and neurological or mental health disorders [13,14]. Having to take many medications at once increases the chance of harmful drug reactions and makes it harder to treat oral health issues. The elderly are more at risk because aging changes in the body, such as less saliva, a weaker immune system, and reduced manual coordination, make oral side effects from medications worse [15].

Polypharmacy can cause several oral health problems, such as a decrease in saliva, trouble with taste, and increased chances of getting opportunistic infections [16]. Furthermore, some medications can cause blood to clot more slowly, increasing the risk of bleeding during dental work, and cognitive or motor problems may make it hard for patients to care for their teeth, raising their chances of getting gum disease. All of these factors have an effect on eating, swallowing, and talking, which can reduce a person’s nutritional health and quality of life [17].

Because polypharmacy can be complicated, dealing with these oral problems requires cooperation among physicians, pharmacists, dentists, and caregivers. Taking care to review medications and offer customized dental care, such as fluorides and substitute saliva, helps reduce oral health issues [18]. Using multiple medications at the same time can cause drugs to interact, resulting in more oral side effects such as dry mouth and easy bleeding. Because of these factors and the usual cognitive and motor problems in elderly patients, oral hygiene may be neglected, and the risk of dental caries and periodontal disease rises [19]. The problems that develop in the mouth can reduce nutrition, make it harder to communicate, and lower the quality of life [20]. Managing these issues calls for teamwork among medical staff and preventive dental care that is suited to the elderly. For a clear picture of common drugs and their oral effects, check Table 1, which organizes the main drug classes, their ways of harming the mouth, and how to manage them.

**Table 1:** Common Systemic Medications and Their Oral Health Effects in Geriatric Patients ACE: angiotensin-converting enzyme; SSRI: selective serotonin reuptake inhibitor; TCA: tricyclic antidepressant; DOAC: direct oral anticoagulant; INR: international normalized ratio

Medication Class	Examples of Drugs	Oral Adverse Effects	Mechanism of Action	Clinical Considerations	References
Antihypertensives	Calcium channel blockers (e.g., nifedipine), ACE inhibitors	Gingival overgrowth, xerostomia	Fibroblast stimulation, salivary gland suppression	Regular dental cleaning; monitor for gingival changes	Marino et al., 2015 [21]
Anticholinergics	Tricyclic antidepressants, bladder antispasmodics, antihistamines	Xerostomia	Block parasympathetic stimulation of salivary glands	Saliva substitutes, hydration, and oral hygiene	Bianco et al., 2021 [3]
Bisphosphonates	Alendronate, zoledronic acid	Medication-related osteonecrosis of the jaw (MRONJ)	Inhibition of osteoclast-mediated bone remodeling	Pre-treatment dental evaluation; avoid invasive dental procedures	Mirhosseini et al., 2024 [15]
Antidepressants	SSRIs (e.g., fluoxetine), TCAs (e.g., amitriptyline)	Xerostomia, taste disturbances	Anticholinergic effects, altered taste receptor function	Monitor for dry mouth; encourage oral hygiene	Janto et al., 2022 [17]
Immunosuppressants	Cyclosporine, tacrolimus, azathioprine	Oral ulcerations, mucositis	Immune suppression leading to mucosal damage and infection	Frequent oral exams; topical corticosteroids	Langari et al., 2022 [9]
Anticoagulants	Warfarin, DOACs (dabigatran, rivaroxaban)	Increased bleeding risk	Inhibition of clotting pathways	INR monitoring (warfarin); local hemostatic measures	Bots-VantSpijker et al., 2021 [6]
Chemotherapy agents	Various cytotoxic drugs	Oral mucositis, ulcerations	Cytotoxic damage to basal epithelial cells	Preventive oral care; symptom management	Desoutte et al., 2012 [22]
Gastrointestinal drugs	Proton pump inhibitors, antacids	Taste disturbances, mucosal atrophy	Altered oral/gut microbiome, salivary changes	Monitor for candidiasis; hydration	Khanagar et al., 2021 [16]

Antihypertensive medications: impact on oral health

Older adults are more likely to have hypertension, which usually requires them to take medications long term [23]. The class of antihypertensive drugs consists of angiotensin-converting enzyme (ACE) inhibitors, calcium channel blockers, beta-blockers, diuretics, and angiotensin receptor blockers (ARBs) [24]. ACE inhibitors and calcium channel blockers are well known for causing oral health problems in elderly patients, which can have serious consequences for their care. They work by stopping angiotensin I from turning into angiotensin II, which helps lower blood vessel constriction and the amount of aldosterone released [25]. Although these drugs are very good at controlling blood pressure, they can cause unpleasant changes in taste and a cough that may make it harder to enjoy food. But calcium channel blockers usually affect oral tissues more seriously than statins do. Many studies have found that calcium channel blockers, which are commonly given for hypertension and angina, can cause the gums to grow excessively [26]. The condition happens when the gums’ connective tissue grows uncontrollably, causing them to swell and become inflamed, making it hard to clean your mouth. The percentage of people on calcium channel blockers who develop gingival overgrowth can be anywhere from 10% to over 50%, depending on how much medication is taken, how long it is taken for, oral hygiene, and the person’s susceptibility [27]. Calcium channel blocker-induced gingival hyperplasia happens because fibroblasts are altered, the extracellular matrix is increased, and inflammation is present. An enlarged gum can result in deeper periodontal pockets, which can trap plaque and make periodontitis more likely [28]. Because of bleeding, discomfort, and poor appearance, some patients may not follow their treatment plans and suffer from poor oral health.

Antihypertensive drugs that are anticholinergic or diuretic can lead to xerostomia in addition to gingival changes [22]. Dry mouth in hypertensive patients adds to the difficulty of treating oral problems because saliva is necessary for healthy mucous membranes and to control bacteria. Together, dry mouth and gum overgrowth make it difficult for doctors to treat elderly hypertensive patients. Treating these mouth problems requires cooperation among medical and dental professionals. Improving oral hygiene and having regular professional cleanings can prevent gingival enlargement. If the tumor grows very large, surgery may be required. If possible, another antihypertensive medicine with a lower risk of oral side effects may be prescribed instead of the calcium channel blocker [29]. Also, using saliva substitutes, staying hydrated, and medicines that help the salivary glands can make patients more comfortable and improve their oral health. As a result, calcium channel blockers and ACE inhibitors are important for treating heart disease in seniors, but they can cause many oral side effects. It is important to notice these side effects and act early to reduce their harm and support good oral health in this group.

Antidepressants and antipsychotics: impact on oral health in the elderly

Many older adults are given antidepressants and antipsychotics to help with a range of psychiatric, neurological, and behavioral issues. Most of these medications are selective serotonin reuptake inhibitors (SSRIs), tricyclic antidepressants (TCAs), or atypical antipsychotic drugs [30]. While these drugs are helpful for some, they often cause dry mouth, teeth grinding, and changes in taste, which can harm the mouths of older adults. Due to their good safety and tolerance, SSRIs are now the main treatment for depression [31]. Still, xerostomia is commonly reported by people who take SSRI medicines. The dryness is caused by anticholinergic effects on the salivary glands, which lower the amount of saliva produced. Saliva helps keep the mouth healthy by fighting bacteria, washing away debris, neutralizing acids, and making the mouth easier to move. A decrease in saliva flow puts patients at risk for dental caries, mouth inflammation, oral thrush, and swallowing and speech difficulties. Since aging and taking multiple drugs often cause dry mouth in older people, SSRI treatment can make these problems worse. TCAs, including amitriptyline and nortriptyline, are recognized for causing severe dry mouth because they are strongly anticholinergic [32]. TCAs might also lead to changes in taste and higher chances of dental and gum disease due to the dry mouth they cause. Because TCAs are used for neuropathic pain and depression that does not respond to other drugs in the elderly, knowing about these oral side effects is important for both clinicians and dentists.

Many doctors prescribe atypical antipsychotics, for example, risperidone, olanzapine, and quetiapine, for psychosis, behavior problems in dementia, and bipolar disorder [33]. These medications are associated with several oral health challenges. Bruxism, or the habit of grinding or clenching teeth without knowing, can result in temporomandibular joint disorders, wear on the teeth, sensitivity, and even cracking. Bruxism in people taking antipsychotics is connected to changes in dopamine and serotonin in the brain. Moreover, some atypical antipsychotics can cause dry mouth, which makes oral problems and infections more likely [34]. Many people using antidepressants and antipsychotics report problems with taste, which can affect both their appetite and their diet. Such disturbances in the senses can make it unpleasant for older adults to eat, leading to malnutrition, which is a major problem for their health.

**Figure 1:**
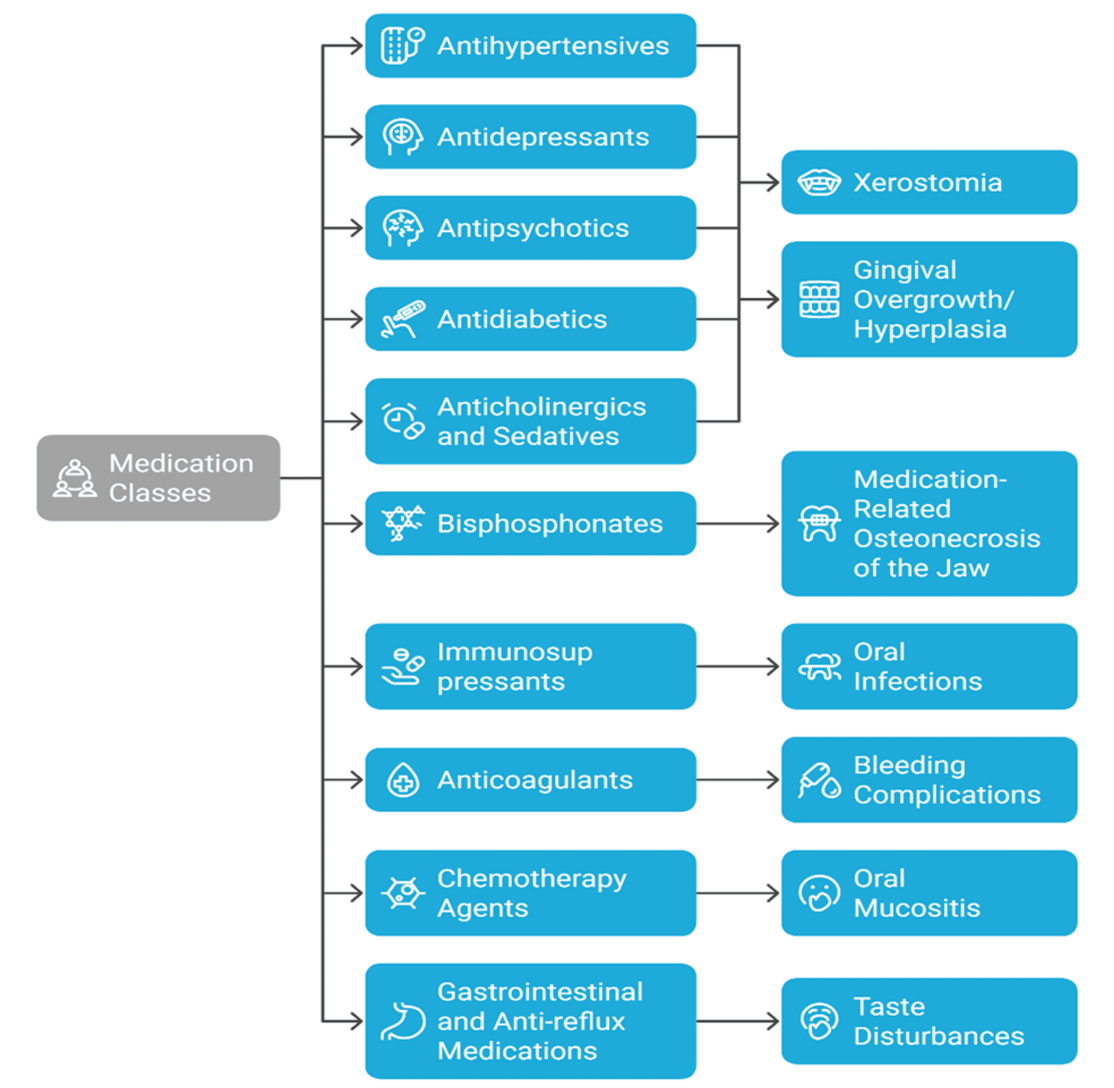
Relationship Between Common Systemic Medication Classes Prescribed to Geriatric Patients and Their Primary Adverse Oral Health Effects Image credit: Avina Kharat

Clinical management of these side effects involves interdisciplinary collaboration. Switching to drugs that cause fewer anticholinergic side effects may improve xerostomia. Reducing complications requires using saliva substitutes, stimulants, good oral hygiene, and regular dental visits [35]. Treatments for bruxism include behavioral therapy, occlusal splints, and occasionally injections with botulinum toxin [17]. In short, antidepressants and antipsychotics are crucial for treating mental health in older adults, but the side effects from taking them orally can be very difficult. It is very important to recognize these problems and use helpful strategies to prevent and treat them in this group. Figure 1 illustrates the main oral health side effects linked to common systemic medications used in the elderly.

Antidiabetics and hypoglycemics: oral health implications in geriatric patients

Many elderly people have type 2 diabetes, which is a common chronic disease, so they rely on antidiabetic medicines such as metformin and insulin [36]. There is a two-way relationship between diabetes and oral health; high blood sugar can damage the gums, while problems in the mouth can make it harder to control blood sugar. In addition, the drugs used to treat diabetes may impact oral health, making the condition worse for elderly patients. Metformin is still the preferred oral medicine for diabetes because it works well, is safe, and provides extra benefits for the heart. While most people tolerate metformin well, a metallic taste and, on rare occasions, stomatitis and lichenoid lesions in the mouth have been linked to the medication [37]. As a result, diabetic patients taking metformin may develop fungal infections in the mouth known as oral candidiasis, since both diabetes and the medication can change the immune system and saliva. Insulin therapy, which is often needed for elderly patients with uncontrolled diabetes, can affect oral health. Although insulin does not harm oral tissues, its presence in treatment often points to more serious health problems that increase the risk of oral problems. High blood sugar levels hinder neutrophil work, reduce collagen formation, and slow wound healing, which increases the risk of periodontal disease, infections, and a slow recovery for people with diabetes after dental treatments [38].

Uncontrolled blood sugar levels have been shown to cause more severe and more common cases of periodontal disease, which damages the structures that hold teeth in place. Periodontitis can make systemic inflammation worse, which may make it harder for people with diabetes to control their blood sugar. In addition, diabetic patients often experience xerostomia, which can be made worse by certain diabetes medications and taking several drugs at once [11,39]. It is difficult to treat diabetic patients with extractions, periodontal surgery, or implants due to the slow healing of oral wounds. Because the body regenerates less well, it is important to plan treatment properly, keep blood sugar under control, and monitor the patient closely after surgery to prevent infections and osteonecrosis.

Oral discomfort and changes in the mouth are common in elderly diabetics with nutritional deficiencies, which complicates their condition. In addition, diabetes can change how the mouth senses pain, which may cause ulcers and other problems to go unnoticed [2]. Management strategies are designed to help people control their blood sugar by taking medicine, making healthy changes in their lifestyle, and having regular check-ups. Looking at oral health, thorough periodontal care, education on how to care for teeth, antifungal treatment when needed, and the use of saliva substitutes can help avoid complications. Endocrinologists, dentists, and primary care providers need to work together to improve health outcomes. Overall, metformin and insulin are important for diabetes management in elderly people, but diabetes also strongly affects the health of their teeth and gums. It is very important to notice and treat the mouth problems and complications that come with diabetes and its treatment to improve the quality of life and lower the risk of sickness in this group (Table 2).

**Table 2:** Clinical Considerations and Dental Management Strategies for Elderly Diabetic Patients on Antidiabetic Medications

Clinical Issue	Underlying Cause	Dental Implications	Recommended Management Strategies	References
Xerostomia	Medication side effects, polypharmacy	Increased risk of caries, mucosal discomfort	Use saliva substitutes, encourage hydration, and apply topical fluoride	Bida et al., 2025 [40]
Delayed wound healing	Hyperglycemia impairs collagen synthesis	Longer recovery from extractions, surgeries	Optimize glycemic control, gentle surgical techniques, and close follow-up	Lelyana et al., 2025 [41]
Increased susceptibility to infection	Immunocompromise, altered saliva composition	Higher risk of candidiasis, periodontal infections	Antifungal treatments, stringent oral hygiene protocols	van der Putten and de Baat 2023 [26]
Taste disturbances	Metformin and other drug effects	Reduced appetite, nutritional challenges	Patient education, dietitian referral if needed	Borg-Bartolo et al., 2025 [8]
Neuropathic oral pain and ulcerations	Diabetic neuropathy	Risk of unnoticed oral trauma and ulcers	Regular oral exams, neuropathy assessment, and protective oral care	Andersson and Kragh Ekstam, 2021 [10]
Periodontal disease exacerbation	Chronic inflammation is linked with diabetes	Increased tooth loss and systemic complications	Intensive periodontal therapy, frequent maintenance visits	Rai et al., 2021 [31]

Anticholinergics and sedatives: effects on oral health in the elderly

Many older adults are given anticholinergics and sedatives to help with urinary incontinence, Parkinson’s disease, psychiatric disorders, and sleep problems. Many of these drugs reduce saliva production by either stopping parasympathetic nerve signals to the salivary glands or by depressing the central nervous system [42]. The most common oral problem caused by these drugs is dry mouth, which can seriously harm the mouth, especially in older people whose saliva production is already reduced. Saliva aids in the health of the mouth by killing bacteria, neutralizing acid, and keeping the mouth lubricated for smooth speech, swallowing, and digestion [43]. A lower level of saliva makes a person more likely to get dental caries, infections of the mouth such as candidiasis, periodontal disease, and mucosal ulcers. People with xerostomia usually feel oral pain, burning, have problems swallowing, notice changes in taste, and are more likely to suffer from mucosal injury. In older people, this problem may also influence their nutrition and daily life.

Having several anticholinergic drugs in your system, such as TCAs, antihistamines, bladder antispasmodics, and some antipsychotics, can increase your risk. Benzodiazepines can make dry mouth worse, as they cause people to become sedated and possibly breathe through their mouths, which reduces fluid intake [44]. As a result of these factors, oral hygiene is more difficult and leads to more plaque and a higher risk of periodontal disease. Managers of anticholinergic drugs should look at medications and try to reduce their anticholinergic effects by using the smallest effective amounts. Treating the symptoms of dry mouth means using saliva substitutes, pilocarpine, drinking often, and practicing good oral hygiene. Patients should avoid tobacco and alcohol, which exacerbate dryness. Regular dental visits help find and treat caries and infections early [26]. Teaching patients how to cope is very important for their comfort and to avoid complications.

Bisphosphonates and osteonecrosis: dental considerations in geriatric patients

Bisphosphonates are often prescribed as powerful antiresorptive drugs for osteoporosis, Paget’s disease, and bone metastases. Using these drugs helps strengthen bones and lowers the risk of fractures, but they can cause a rare and serious condition called MRONJ, which is exposed necrotic bone in the jaw that does not heal within eight weeks [45]. Because elderly patients receive the most bisphosphonate therapy, this adverse effect is a big problem for them. MRONJ develops when osteoclast activity is reduced, which leads to bone remodeling problems, a lack of new blood vessels, and an increased risk of infection after dental procedures or injury [7]. The dense bone and poor blood flow in the mandible make it the most common site for osteomyelitis. Risks are greater when intravenous bisphosphonates are used for cancer, but they also exist with long-term use of oral bisphosphonates to treat osteoporosis.

Exposed bone, with or without pain, swelling, infection, or a fistula, is the main feature of MRONJ in clinical practice. Often, it happens after dental extractions, implant placements, or other invasive procedures, but it can also happen without warning [5]. Management is not easy and usually involves rinsing with antimicrobials, using antibiotics, controlling pain, and surgically removing dead tissue in severe cases. Dental care in bisphosphonate-treated elderly patients should prioritize prevention. Treating active infections and evaluating the mouth should be done before starting bisphosphonates whenever possible. Taking care of your mouth and not having unnecessary dental work during treatment helps prevent MRONJ. If dental surgery cannot be avoided, it should be done with the least amount of trauma and with antibiotic prophylaxis. For the best care, dental and medical teams must cooperate [46]. As a result, bisphosphonates are essential for treating bone problems in the elderly, but their link to osteonecrosis of the jaw means doctors have to watch carefully. Identifying problems early, preventing them, and planning dental care well are important for reducing health risks and keeping the mouth healthy in this group.

Immunosuppressants and oral ulcerations: clinical implications in geriatric patients

People with transplants and autoimmune diseases, which are common in the elderly, are often helped with immunosuppressant medications. To stop the body from rejecting the transplanted organ or to control unusual immune responses, these drugs, such as corticosteroids, calcineurin inhibitors (cyclosporine, tacrolimus), and antimetabolites (azathioprine, mycophenolate mofetil), are used to suppress the immune system. But because of immunosuppression, patients are more likely to get painful oral ulcers, for example, oral lichen planus (OLP) and recurrent aphthous stomatitis (RAS) [47]. Oral ulcers in these cases can be caused by several different factors. Taking drugs can cause direct damage to mucosal cells, and immune suppression makes it harder for the body to control infections and inflammation, which keeps the mucosa injured. OLP is an ongoing immune condition that often affects those with weakened immune systems, causing white, reticular, or erosive lesions on the buccal mucosa, tongue, and gingiva [36]. Erosive forms are very painful and increase the risk of both infections and cancer. It is difficult to manage patients with HIV because the immune system can cause new lesions and slow down healing.

**Figure 2:**
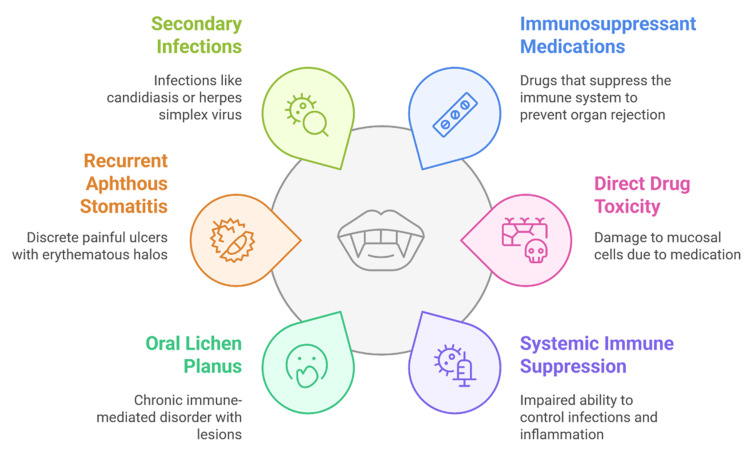
Key Factors Contributing to Oral Ulcerations in Geriatric Patients Image credit: Avina Kharat

RAS often appears as painful ulcers surrounded by redness, and the condition is usually made worse by weak mucosal immunity and other infections such as candidiasis or herpes simplex virus. Taking immunosuppressants can make mucositis, dry mouth, and other infections more likely, which can be uncomfortable for elderly patients and complicate their treatment [48]. Management works best when it is handled by a team of physicians and dental professionals. Regular check-ups and early detection help to identify whether lesions are caused by drugs by infections, or cancers. Doctors treat the condition by easing symptoms, promoting healing of the mucous membranes, and preventing infections with topical corticosteroids, antimicrobials, and antiviral drugs [9]. Patients need to learn about oral hygiene, avoiding irritants, and healthy eating, given that many elderly people are on several medications and have several health conditions. Hence, immunosuppressants are indispensable but increase oral ulceration. Caring for the mouth as part of general health improves the lives and health of these patients. Figure 2 outlines the main reasons why oral ulcers occur in elderly patients. It explains how immunosuppressant drugs help prevent the body from rejecting transplanted organs and, at the same time, lower the body’s ability to fight infections and inflammation.

Cancer chemotherapy and oral mucositis

Anticoagulant therapy should be given to prevent and treat thromboembolic disorders like atrial fibrillation, deep vein thrombosis, pulmonary embolism, and stroke, which often affect the elderly. Among anticoagulants, warfarin is traditional, and newer direct oral anticoagulants (DOACs) include dabigatran, rivaroxaban, apixaban, and edoxaban [49]. While they help prevent blood clots, these drugs raise the risk of bleeding, which can be problematic during dental and periodontal treatments. Doctors must ensure that elderly patients on anticoagulants are not at risk for bleeding and also for thromboembolism if they miss a dose. Problems such as delayed healing, infection, and hospitalization may occur if too much bleeding happens during dental extractions, implant placement, or soft tissue surgeries [21]. The international normalized ratio (INR) is used to check warfarin’s anticoagulant effect, and dental care is usually safe if the INR is below 3.0. Stopping warfarin for minor dental surgery is generally not recommended, unless the INR is very high or the surgery is major.

Even though DOACs have less risk of interactions, their effects are easier to predict, but there is no routine way to check for bleeding. Because they only last a short time in the body, they can be stopped 24-48 hours before an invasive procedure to control the risk of bleeding and clotting [45]. Problems related to bleeding usually involve prolonged bleeding, hematomas, or mucosal bleeding, which are generally controlled with local pressure, sutures, topical hemostatics, and tranexamic acid rinses. Planning for surgery should take into account any past bleeding, the patient’s current medications, and any health problems that can cause bleeding. Patients need to learn about post-procedure care, and doctors need to coordinate therapy management. Anticoagulants may cause the gums to bleed, which can make periodontal treatment difficult and discourage patients from seeing the dentist [37]. For dental care to be safe for elderly patients who take anticoagulants, it is important to use a multidisciplinary approach, check the risks, and plan carefully.

Cytotoxic effects and oral hygiene challenges

Many types of cancer, including those found in the elderly, are treated with chemotherapy. Chemotherapy works well to treat cancer, but it also damages normal cells in the oral mucosa that divide rapidly [50]. Oral mucositis is a common and serious side effect in the mouth, marked by inflammation, redness, ulcers, and pain of the mucosa [27]. As a result, oral function is greatly reduced, nutrition is affected, and the risk of both local and general infections rises. Chemotherapy-induced oral mucositis is caused by a variety of biological events that start when DNA in basal epithelial cells is damaged. This causes reactive oxygen species to form, turns on transcription factors like nuclear factor-kappa B (NF-kB), pushes out pro-inflammatory cytokines, and results in tissue damage and ulceration [33]. When the mucosal barrier is disrupted, bacteria can easily get inside and cause more infections, which usually makes symptoms worse and takes longer to heal. Both aging and other health problems in geriatric patients make mucositis more severe and last longer.

Oral mucositis is first seen as redness in the mouth, then develops into painful ulcers that make it difficult to chew, swallow, or talk. Because the pain and discomfort make it hard to eat, these patients often lose weight, which can lead to malnutrition and poorer results from treatment [51]. Besides, mucositis often leads to chemotherapy dose cuts or pauses in treatment, which can affect how well the cancer is controlled. Management of oral mucositis requires a multidisciplinary approach. Prevention approaches include using oral cryotherapy, low-level laser therapy, and giving palifermin and similar keratinocyte growth factors, which have been proven to decrease the number and severity of cases [10]. It is very important to keep the mouth clean to avoid further infections, but mucositis makes it difficult to take care of the mouth because of the pain and how easily the mouth becomes irritated.

**Figure 3:**
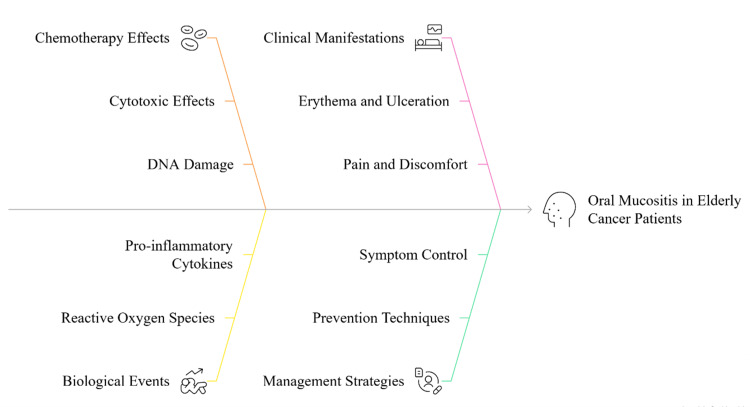
Factors Contributing to Oral Mucositis in Elderly Cancer Patients Image credit: Avina Kharat

Symptom control requires using non-irritating mouth rinses, topical anesthetics, and systemic analgesics. In severe cases, it may be important to change the patient’s diet and use enteral feeding. Antimicrobial therapy should be used when secondary infections develop [44]. Routine dental check-ups before, during, and after chemotherapy allow early detection and treatment of mucositis and other mouth problems. Geriatric patients require special care since taking multiple drugs, having multiple health problems, and being less mobile can all make oral hygiene practices more complicated. Helping caregivers with education and support is important for making sure patients follow their oral care routines [52]. In general, reducing the effects of oral mucositis helps maintain a good quality of life and successful treatment for elderly cancer patients. Figure 3 provides an overall view of the different factors that contribute to the development and management of oral mucositis in elderly patients receiving cancer chemotherapy.

Gastrointestinal and anti-reflux medications: oral side effects and clinical implications

Among the elderly, gastrointestinal disorders, particularly gastroesophageal reflux disease (GERD), are quite common and usually need long-term use of medication [25]. Doctors frequently give proton pump inhibitors (PPIs) and antacids to lower gastric acid and ease symptoms of reflux [24]. Although these medicines are usually considered safe, new research suggests that they can trigger taste changes and mucosal problems in the mouth, which may affect a patient’s well-being. PPIs stop the hydrogen-potassium ATPase enzyme in gastric parietal cells, which in turn reduces acid production. However, taking PPI for a long time can change the bacteria in the mouth and intestines because the pH and numbers of bacteria are affected. As a result of such dysbiosis, patients may be more likely to develop oral candidiasis and other infections that take advantage of weak immune systems, mainly in the elderly.

It has also been noticed that PPIs and antacids can cause people to have a metallic, bitter, or reduced sense of taste [11]. When taste changes, older people may eat less, get fewer nutrients, and experience a lower quality of life, since they are already at risk for malnutrition. Experts are not sure how these taste changes occur, but they might involve changes in our saliva, effects on taste receptors, or changes in the way our digestive organs function. Dry mouth and atrophic mucosal changes caused by these drugs can worsen current oral problems or lead to new discomfort in the mouth [23]. Acid suppression therapy can lower the acidity in the body, which may reduce the uptake of minerals and harm the mouth’s bone health, possibly leading to periodontal disease or delays in healing after dental work.

Doctors should keep these possible side effects in mind when giving long-term gastrointestinal medications to elderly people. When doing an oral examination and taking a patient’s history, make sure to ask about changes in taste and mucosal symptoms. We should advise patients to brush their teeth, drink enough water, and keep an eye out for infections like candidiasis [32]. Good communication among dentists, gastroenterologists, and primary care physicians is essential for the best care of patients. If possible, drug regimens should be reviewed to avoid oral problems without reducing treatment for the gut. Other therapies or treatments might be tried, and saliva substitutes and topical products can be used to ease the discomfort (Table 3).

**Table 3:** Oral Side Effects Linked to Gastrointestinal and Anti-reflux Medications in Elderly Patients

Oral Side Effects	Associated Medication(s)	Risk Factors	Clinical Impact	Management Recommendations	References
Taste disturbances (dysgeusia)	Proton pump inhibitors (PPIs), antacids	Long-term use, polypharmacy	Reduced appetite, nutritional risk	Monitor taste changes, dietary counseling, and assess medication necessity	Desoutter et al., 2012 [22]
Xerostomia and dry mouth	PPIs, antacids	Dehydration, coexisting medications	Oral discomfort, increased caries risk	Encourage hydration, use saliva substitutes, and maintain oral hygiene	Bots-VantSpijker et al., 2022 [14]
Oral candidiasis	PPIs	Immunosuppression, poor hygiene	Mucosal soreness, infection	Antifungal therapy, oral hygiene reinforcement	Fornari et al., 2021 [5]
Mucosal atrophy	Long-term acid suppression therapy	Nutritional deficiencies, aging	Increased mucosal fragility	Regular oral exams, topical lubricants	Ástvaldsdóttir et al., 2018 [37]
Delayed mucosal healing	Acid suppression agents	Comorbidities, dental surgery	Prolonged recovery from procedures	Pre- and post-operative care, optimize systemic health	Marino et al., 2015 [21]
Periodontal disease progression	PPIs and associated mineral absorption impairment	Poor oral hygiene, systemic inflammation	Loss of attachment, increased tooth mobility	Frequent periodontal maintenance, mineral supplementation assessment	Rosa et al., 2020 [29]

Limitations of the current evidence

Even though more people are aware of how systemic medications can harm oral health, the current research still has some major limitations. A key problem is that there are not many randomized controlled trials (RCTs) that study oral health outcomes linked to taking systemic drugs. Most of the research done so far is observational or retrospective, so it is hard to know the exact causes or how common oral problems caused by medicines are [5]. Often, geriatric patients are not included in these studies because of their many health problems and the use of several medications. Without age-specific data, it is difficult to apply the findings to the elderly, who have special weaknesses that can make oral side effects worse.

Since people in different regions and with different diets respond differently to drugs and oral health care, it is difficult to compare populations. In addition, many patients do not report dry mouth or taste problems, or they blame these problems on aging instead of thinking they are caused by their medications. Because this condition is not well recognized, clinical trials and pharmacovigilance data are incomplete, which leads to gaps in epidemiology and no standard guidelines for managing it. To address these gaps, we should conduct geriatric-focused research and standardize oral assessments to help find and control oral health problems caused by medications in older adults.

Future recommendations

Elderly patients on long-term medications often have complex oral health problems, requiring a combination of strategies to address them effectively. All healthcare professionals, including geriatricians, dentists, and pharmacists, should be encouraged to cooperate to provide care that looks after the patient’s oral health and overall medical issues. Having standardized geriatric oral health screening protocols helps identify medication-related oral problems early, so treatment can be given promptly and the outcome can be better. Also, including specific oral health lessons in geriatric training will help clinicians better handle these problems. With so many patients on several medications, research should focus on ways to prevent common dental problems, including dry mouth, mouth lesions, and other side effects. Additionally, there is a strong requirement for research that follows the development and consequences of drug-related oral problems over the years, so that reliable data can be used for developing clinical guidelines. All of these recommendations are designed to encourage active, preventive, and patient-focused oral care for the elderly, which should lead to a better quality of life and a lower risk of oral diseases caused by medications.

## Conclusions

Systemic medications that are often used in geriatric patients, including antihypertensives, antidepressants, antidiabetics, anticholinergics, bisphosphonates, immunosuppressants, anticoagulants, chemotherapy, and gastrointestinal medications, have numerous adverse effects on oral health. The adverse effects of these drugs on oral health range from xerostomia to gingival hyperplasia, ulcerations of the mucosa, osteonecrosis of the jaw, bleeding complications, taste disturbances, and infection susceptibility. The multifactorial etiology of these oral findings, frequently augmented by polypharmacy and physiological changes inherent in aging, highlights the complexity of oral health management in older populations. Since oral health plays such a crucial role in nutrition, communication, and quality of life, these drug-induced side effects have important clinical and social ramifications that warrant special attention.

The geriatric patient's susceptibility to oral adverse effects is compounded by the presence of several comorbid conditions and frequent intake of multiple medications. This calls for an integrated method that encourages teamwork between medical and dental health caregivers for proper assessment, prevention, and management of oral complications. Raise awareness among clinicians and caregivers regarding oral consequences of systemic treatments, as it is crucial to early detection and timely intervention. In the end, a multidisciplinary, patient-based approach that closes the gap between general medical and dental treatment will enhance health status and quality of life among older patients confronted with complicated medication regimens.
